# Towards an adult hearing screening procedure

**DOI:** 10.1016/j.bjorl.2025.101616

**Published:** 2025-04-11

**Authors:** Marc Jan-Willem Lammers, Chris Raine, Griet Mertens, Vincent van Rompaey, Rudolf Hagen, Anja Kurz, Piotr Henryk Skarzynski, Artur Lorens, Jane Opie, Patrick D’Haese, Peter Grasso, Luis Lassaletta, Miryam Calvino, Ilona Anderson

**Affiliations:** a Antwerp University Hospital (UZA), Department of Otorhinolaryngology Head and Neck Surgery, Edegem, Belgium; b University of Antwerp, Faculty of Medicine and Health Sciences, Department of Translational Neurosciences, Resonant Labs Antwerp, Wilrijk, Belgium; c Bradford Royal Infirmary Yorkshire Auditory Implant Center, Bradford, United Kingdom; d University Hospital Wuerzburg, Comprehensive Hearing Center, Department of Otorhinolaryngology, Head and Neck Surgery, Würzburg, Germany; e Institute of Physiology and Pathology of Hearing, World Hearing Center, Kajetany, Warsaw, Poland; f MED-EL GmbH, Innsbruck, Austria; g MED-EL GmbH, Department of Awareness and Public Affairs, Innsbruck, Austria; h La Paz University Hospital, IdiPAZ Institute for Health Research, Department of Otolaryngology, Madrid, Spain; i Institute of Health Carlos III, Biomedical Research Networking Centre On Rare Diseases (CIBERER-U761), Madrid, Spain; j Clinical Research Department, MED-EL GmbH, Innsbruck, Austria

**Keywords:** Screening, Prevention, Hearing loss, Cognition, Dementia

## Abstract

**Objective:**

The first aim was to develop a simple, yet effective adult hearing screening protocol, based on the existing World Health Organization recommendations. Secondary aim was to gain insight in the possibilities and potential obstacles for implementation of national adult hearing screening programs across the globe.

**Methods:**

An expert working group was formed with 14 experts from six countries. Based on their recommendations a HEARRING screening protocol was devised and shared with all HEARRING group members. A survey was sent out to 62 clinicians from 22 countries to hear their considerations and the potential challenges they expect for the introduction of national adult hearing screening programs.

**Results:**

The proposed adult hearing screening protocol is made as simple and least time consuming as possible. The tablet-based screening tool consists of two red flag questions, followed by the Hearing Handicap Inventory for the Elderly Screening (HHIE-S) questionnaire and the Digits-in-Noise test, and is developed to be implemented in the primary care setting. The results of our survey indicate that most participants deem a national adult hearing screening to be cost-effective, but the major concerns regarding costs and funding, and infrastructure have to be discussed.

**Conclusion:**

The HEARRING screening protocol is developed to assist clinicians and policy makers in their efforts to initiate effective local and national adult hearing screening programs.

## Highlights

• The adult HEARRING screening protocol is a consensus protocol.
• It is developed for easy implementation in primary care clinics.
• The protocol is based on the World Health Organization recommendations.
• It can assist clinicians in initiating effective hearing screening programs.

## Introduction

With the aging population, hearing loss is an increasing public health concern. It is estimated that two thirds of all adults over 60-years of age have some degree of hearing loss.^1^ Progressive age-related hearing loss not only affects speech understanding and daily communication, but also social functioning. In the past two decades there is accumulating evidence indicating that untreated hearing loss is associated with a higher risk of cognitive decline and dementia.^1^, ^2^, ^3^, ^4^ In 2020, and again in their 2024 update, the Lancet Commission on Dementia concluded that hearing loss is the most important modifiable risk factor for dementia.^5^, ^6^ Their conclusions and recommendations are in line with the 2017 Integrated Care for Older People (ICOP) guideline of the World Health Organization (WHO), which also states that hearing screening and hearing care should be an integral part of the primary care for the elderly.^7^ The recent ACHIEVE trial confirmed the benefits of hearing aid use on communication, and also revealed that it can reduce the cognitive decline in especially those who are at risk for developing dementia.^3^ Despite all strong recommendations, hearing screening programs for older people are not in place yet. In 2021 the US Preventive Services Task Force (USPSTF) concluded that there is insufficient evidence to support general hearing screening in adults.^8^ However, potential secondary cost and quality of life savings of dementia prevention by the implementation of hearing screening were not analysed. Given the positive effects of hearing aid and cochlear implant use on quality of life and cognitive decline in older adults, indirect cost savings might pave way for general hearing screening programs and improved access to hearing aids and cochlear implants.^3^, ^9^, ^10^

Over the years, the number of hearing screening initiatives for adults has been growing. Most studies on hearing screening initiatives have used self-administered telephone or web-based hearing screening tests which had to be accessed from home.^11^, ^12^, ^13^ The home setting has detrimental negative effect on the completion rate of such tests as recently demonstrated by Smith et al. 2023. In their study with 660 adults, they found that only 26.8% of the patients completed the screening test at home, whereas all the participants who were asked to perform the telephone screening test at the primary care clinic completed the screening.^14^ Technological advancements have improved the user-friendliness and availability of newer web and application based screening tools. Recently the WHO launched the HearWHO app, using the digits-in-noise test which is available in a growing number of languages.^15^ These new screening tools are promising for future hearing screening programs, but administration of the screening tests during physical appointments at primary care facilities seem to be still required to guarantee high completion rates and to improve the cost-effectiveness.

In order to construct an effective, but also generic screening approach suitable for different global regions, an expert working group was formed. The first aim of this working group was to discuss the WHO guidelines and recommendations, to simplify their screening proposals and to optimize them for integration in busy, daily clinical settings. Second, to increase the likelihood of successful implementation and to assure integration into different healthcare systems, the opportunities and obstacles for screening were assessed by sending out questionnaires to clinicians in 22 countries.

## Methods

A working group was formed consisting of fourteen clinicians, audiologists and otorhinolaryngologists, from six European countries. Three meetings were held to discuss the local and national opportunities to initiate an adult hearing screening program. Questions regarding how and where to screen, and for which target population were discussed and consensus recommendations were made. The hearing screening flow chart proposed by the WHO was evaluated by the working group with the aim to optimize the screening flow for easy clinical use.

After the consensus recommendations were made, an online survey was established. The survey consisted of eight questions, listed in Table 1, covering topics like the potential (cost)-effectiveness of adult hearing screening programs, and expected obstacles which might hinder implementation of such screening. The online survey was sent out to 62 clinicians, from 36 HEARRING clinics from 22 countries.

**Table 1:** Survey with eight questions.

**Question**		**Answer**
1.	Do you expect that in your country/region a tablet based self-administered adult hearing screening program can be implemented in primary care facilities?	Yes
		Yes, but only if it does not require additional time of staff or costs
		Perhaps, but there are still several obstacles to overcome
		No
2.	Do you expect that in your country/region an adult hearing screening program will be organized in the future by the government?	Yes
		No, due to financial reasons
		No, due to a lack of interest
3.	Do you expect that in your country/region the government will co-finance a joint effort with non-profit partners to introduce an adult hearing screening program?	Ye
		Perhaps
		No
4.	Do you think that in your country/region an adult hearing screening program can become cost-effective (from ENT/audiologist perspective)?	Yes
		Perhaps
		No
5.	Do you expect that healthcare policy makers in your country/region will judge an adult hearing screening program to be cost-effective?	Yes
		Perhaps
		No
6.	Does your country have guaranteed adequate access to treatment after a referral? (i.e., local ENTs and hearing aids)	Yes
		Access to follow up care is good, but hearing aid reimbursement is limited
		Access to follow up is limited, but hearing aid reimbursement is good
		For the majority of the population, both access and hearing aid reimbursement are insufficient
7.	Do you think that in your country hearing care is a top priority for healthcare policy makers or funding organizations?	Yes/No
8.	Which three major obstacles do you foresee in your country/region for implementation of an adult hearing screening program?	Open

## Results

### Consensus on adult hearing screening program

The first recommendation by our working group was that the screening program should be simple and self-explanatory, easy to perform by the target population, and little time consuming for both patient and healthcare workers. The recommended target population consists of adults aged 50 years or higher, which is in line with the recommendation by the WHO.

The second recommendation was to perform the hearing screening within the primary care setting. This could be either in the waiting room or during specific screening visits where patients are screened for other conditions, such as cardiovascular risk factors. The decision to perform the screening within the primary care setting was based on the previous experiences with self-administered telephone, and internet hearing screening tests and the recent results by Smith et al. demonstrating that screening within the primary care clinics significantly improves the completion rate.^14^ The exact approach on how to implement the hearing screening in the daily primary care setting is left to the discretion of the primary care clinics.

**Fig. 1:**
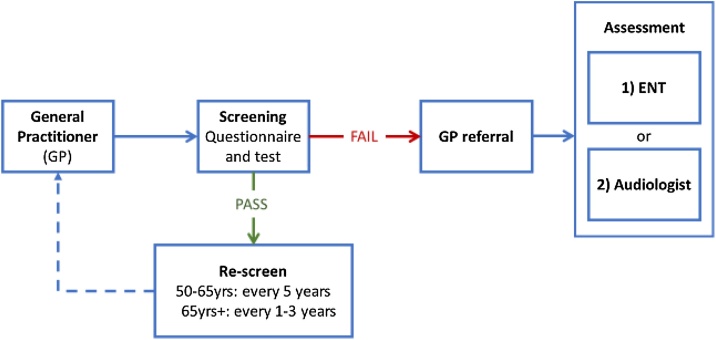
HEARRING screening protocol flow chart. In case of a screening pass, it is advised to screen at least every 5-years in adults between 50- and 65-years; for adults over 65-years in every one to three years. In case of a screening fail, it is advised to perform otoscopic examination to exclude external ear canal or middle ear conditions. Secondary assessment by otolaryngologist or audiologist is dependent on national and regional referral policies and is left to discretion of the general practitioner.

The third recommendation was to simplify the screening flow as proposed by the WHO. The WHO flow chart published in the ICOP guideline was thought to be too complex for use in the very busy primary care setting. After discussion, a new and more simple flow chart was composed, which was considered to be more suitable for clinical practice (Fig. 1). The first adaptation to the WHO flow chart is to perform the screening test regardless of reported subjective hearing loss. In the WHO flow chart, an immediate referral to diagnostic audiology is recommended if subjective hearing loss was reported by the patient (without performing the screening test). Second, the results of both the hearing test and the responses to the screening and red flag questions will be combined for a final pass or fail. In case of a positive answer to the red flag questions, an immediate fail will be given, regardless of the screening test outcome. Finally, the otoscopic examination for wax removal recommended by the WHO is removed from our flow chart, as the impact of wax on hearing thresholds is only mild (5–10 dB) and it unnecessary complicates the screening procedure. It is left to the discretion to the clinician performing the screening test to either remove the wax and repeat the screening or refer to an Ear, Nose and Throat (ENT) clinic.

The proposed screening program will be tablet based and performed within the primary care setting. Support and encouragement by the primary care physicians and nurses will be needed for successful implementation. Posters and flyers, aimed at increasing awareness on the importance of hearing loss, will be available for distribution in the waiting rooms. The screening starts with two questions on red flags which require immediate attention, such as symptoms of ear infections. (Do you experience ear discharge, ear pain or dizziness? Are you known with unilateral or asymmetric hearing loss, or rapidly deteriorating hearing loss?) The screening is then followed by the Hearing Handicap Inventory for the Elderly Screening (HHIE-S) and finally by the Digits-in-Noise test.^16^, ^17^, ^18^, ^19^ At the end of the screening, patients will either receive a pass or a fail. In case of a fail the primary care physician can directly refer the patient to an otorhinolaryngologist or audiologist for further examination. In case of a pass, patients are advised to perform re-screen every five years and after the age of 65-years every one to three years. The exact follow up screening frequency is left to the discretion of the primary care clinics.

### Survey

**Fig. 2:**
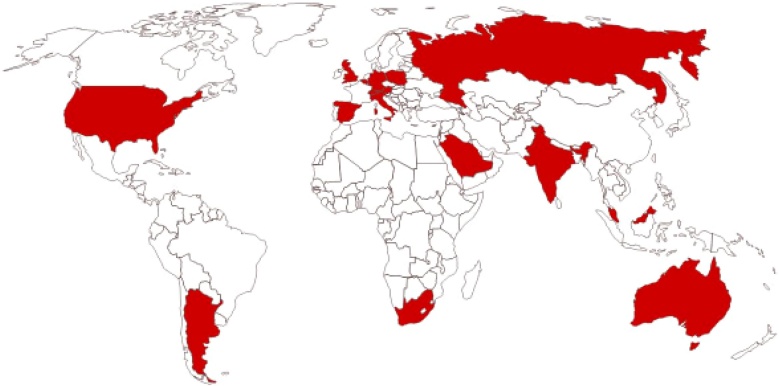
Participating countries.

The online survey was returned by 38 clinicians, yielding a good response rate of 61% (38/62). These 38 participants were spread over 16 different countries and six continents (Fig. 2). The vast majority of the clinicians (76%) had the opinion that hearing care is not a top priority for health care policy makers or funding organizations in their country, despite the various WHO reports. Adult hearing screening programs have been advised by the WHO since 2017, but none of the countries have implemented a screening program yet. The majority of the respondents do not expect their (local) government to initiate an adult hearing screening program due to financial reasons (47%) or lack of interest (26%). On the other hand, most clinicians deem it possible and cost-effective (92%) to implement a tablet based self-administered adult hearing screening program in the primary care setting of their country, if obstacles are overcome. The major obstacles potentially hindering implementation are related to costs, infrastructure, and awareness. Joint initiatives with non-profit partners to introduce a screening program with co-financing by the government, might be an attractive alternative (84%). Besides the cost-effectiveness of screening programs, adequate follow up after screening referral is another essential element. Access to local ENTs is adequate in the majority of the countries (70%) but satisfactory reimbursement for hearing aids is a limiting factor in eleven of the sixteen countries.

## Discussion

The results of our survey included participants from 16 different countries spread over six continents reveal the global need and importance to establish hearing screening programs for the elderly. This need is not only supported by the WHO, but also by the growing evidence indicating that hearing loss is a modifiable risk factor for dementia.^1^ The adult hearing screening programme, as proposed by the working group, was believed to be suitable and cost-effective for almost all of the 16 participating countries. Unfortunately, hearing care in adults does not have a high priority for politicians and policy makers in most of the countries. Despite technological advances in hearing screening tests and mobile applications, concerns regarding costs and infrastructure are still expected to be the major obstacles for realizing a large-scale hearing screening program. Local and regional bottom-up initiatives could therefore pave the way for broader adaptation at the national level.

One of the major obstacles for hearing screening is the uptake in especially the older population. Data from 242.626 hearing screening tests with the hearWHO app revealed the highest uptake of 44% in young adults under the age of 30, in contrast to only a few percent in the target population of over 60.^15^ Screening test uptake has been effectively increased by performing the screening tests during primary care visits.^14^ This emphasizes the importance of creating awareness and informing patients about the consequences of hearing loss, and supports our view to perform the screening within the primary care setting. We advise to initiate a hearing screening initiative in close collaboration with the primary care physicians and provide them with sufficient flexibility to effectively implement the screening in their busy clinics. The exact frequency of follow up screenings after a pass and the coordination of the screening at the local sites, is therefore left to the discretion of the primary care clinics.

The USPSTF recommended in 2021 that there was insufficient evidence to support the cost-effectiveness of adult hearing screenings. Since this publication, more studies revealed the consequences of hearing loss on cognitive decline and the therapeutic benefit of interventions such as hearing aids and cochlear implants on cognition.^1^, ^3^, ^9^, ^10^ If we would consider the potential indirect cost savings realized by early identification and treatment of hearing loss on the lifelong management of the elderly, the balance between screening costs and cost savings could easily tip the other way. The technological innovations will make hearing screening setups not only more user friendly, but also less expensive in operation and maintenance. The vast majority (92%) of our survey respondents share this perspective and expect a national adult hearing screening to be (potentially) cost-effective. Although hearing screening in primary care clinics is more expensive, researchers from Duke University calculated that the quality of life improvements make up for the additional costs.^20^, ^21^ An additional advantage of screening within the primary care setting is the potential to increase the effectiveness of follow up after the screening. It is more likely that with motivation from primary care physicians more patients seek follow up treatment after referral, compared to a home-based screening scenario. Our survey results reveal that the access to follow up at local ENTs is adequate in the majority of the participating countries, but the reimbursement for hearing aids is insufficient and a limiting factor in most countries (85%). This concern is shared by many others and hope is set that with innovations in the hearing aid market, costs will go down improving accessibility.^22^

Another challenge is the aim to develop a universal screening methodology to fit most regions and countries. Although input has been gathered from 16 different countries, it was mainly based on situations in developed countries. Our suggested screening protocol is thus more aimed at health care systems in developed countries with good access to primary and secondary care clinics. As a result, this limits the generalizability of our screening. It is therefore advised to be used as guidance and to be adapted to fit the regional needs. Another limitation is the language dependency of the digits-in-noise test and the HHIE-S questionnaire. Although both are available in a growing number of languages, translating and validating new language versions is time consuming. The development of language independent screening tests, such as Aladdin (Automatic LAnguage-independent Development of the Digits-In-Noise test) may solve these issues and improve global accessibility.^23^

The ambition to establish a simple, fast and effective hearing screening protocol, does also has its ramifications. The screening can indicate the presence of hearing loss, but further details should be explored at appropriate follow up consultations. For example, it does not cover aspects like the type of hearing loss, history of hearing loss and noise exposure, genetics, or cognition. Furthermore, cultural differences and stigmas can have a major impact on the implementation of hearing screening programs. The proposed protocol is therefore only to be used as a guidance, and national and regional adaptations can be made to facilitate implementation and improve the uptake.

## Conclusion

The proposed HEARRING screening protocol is developed to assist clinicians and policy makers in their efforts to initiate effective hearing screening programs in the primary care setting. Although for all countries and regions obstacles need to be overcome, most clinicians in our survey still deem it possible to implement a tablet based self-administered adult hearing screening program in the primary care setting of their country. The major concerns are related to costs, infrastructure, and awareness, which should all be addressed at the regional and national levels. Nevertheless, for the near future, grassroot initiatives are the most likely to be successful. With adequate technical and financial support for the development of screening tests and awareness campaign material, regional initiatives with motivated partners can inspire a more collective and national approach.

## Funding

This research project is supported by the HEARRING Group and MED-EL GmbH. The HEARRING Group is an independent network of world leading centres and experts dealing with all aspects of hearing disorders. The authors of the paper are members of the HEARRING group.

## Declaration of competing interest

The authors declare no conflicts of interest.

## References

[1] Lin F.R. (2024). Age-related hearing loss. N Engl J Med..

[2] Davies H.R., Cadar D., Herbert A., Orrell M., Steptoe A. (2017). Hearing impairment and incident dementia: findings from the english longitudinal study of ageing. J Am Geriatr Soc..

[3] Lin F.R., Pike J.R., Albert M.S., ACHIEVE Collaborative Research Group (2023). Hearing intervention versus health education control to reduce cognitive decline in older adults with hearing loss in the USA (ACHIEVE): a multicentre, randomised controlled trial. Lancet.

[4] Lin F.R., Yaffe K., Xia J. (2013). Hearing loss and cognitive decline in older adults. JAMA Intern Med..

[5] Livingston G., Huntley J., Sommerlad A. (2020). Dementia prevention, intervention, and care: 2020 report of the Lancet Commission. Lancet.

[6] Livingston G., Huntley J., Liu K.Y. (2024). Dementia prevention, intervention, and care: 2024 report of the Lancet standing Commission. Lancet.

[7] Integrated Care for Older People: Guidelines on Community-Level Interventions to Manage Declines in Intrinsic Capacity. 2017. WHO Guidelines Approved by the Guidelines Review Committee. 29608259

[8] Krist A.H., Davidson K.W., Mangione C.M., US Preventive Services Task Force (2021). Screening for hearing loss in older adults: US preventive services task force recommendation statement. JAMA..

[9] Andries E., Bosmans J., Engelborghs S. (2023). Evaluation of Cognitive Functioning Before and After Cochlear Implantation in Adults Aged 55 Years and Older at Risk for Mild Cognitive Impairment. JAMA Otolaryngol Head Neck Surg..

[10] Mertens G., Andries E., Claes A.J. (2021). Cognitive improvement after cochlear implantation in older adults with severe or profound hearing impairment: a prospective, longitudinal, controlled, multicenter study. Ear Hear..

[11] Watson C.S., Kidd G.R., Miller J.D., Smits C., Humes L.E. (2012). Telephone screening tests for functionally impaired hearing: current use in seven countries and development of a US version. J Am Acad Audiol..

[12] Smits C., Merkus P., Houtgast T. (2006). How we do it: The Dutch functional hearing-screening tests by telephone and internet. Clin Otolaryngol..

[13] Meyer C., Hickson L., Khan A., Hartley D., Dillon H., Seymour J. (2011). Investigation of the actions taken by adults who failed a telephone-based hearing screen. Ear Hear..

[14] Smith S.L., Francis H.W., Witsell D.L. (2024). A pragmatic clinical trial of hearing screening in primary care clinics: effect of setting and provider encouragement. Ear Hear..

[15] De Sousa K.C., Smits C., Moore D.R., Chada S., Myburgh H., Swanepoel W. (2022). Global use and outcomes of the hearWHO mHealth hearing test. Digit Health..

[16] Smits C., Kapteyn T.S., Houtgast T. (2004). Development and validation of an automatic speech-in-noise screening test by telephone. Int J Audiol..

[17] Smits C., Theo Goverts S., Festen J.M. (2013). The digits-in-noise test: assessing auditory speech recognition abilities in noise. J Acoust Soc Am..

[18] Humes L.E. (2021). An approach to self-assessed auditory wellness in older adults. Ear Hear..

[19] Weinstein B.E. (1990). The quantification of hearing aid benefit in the elderly: the role of self-assessment measures. Acta Otolaryngol Suppl..

[20] Borre E.D., Dubno J.R., Myers E.R. (2023). Model-projected cost-effectiveness of adult hearing screening in the USA. J Gen Intern Med..

[21] Dubno J.R., Majumder P., Bettger J.P. (2022). A pragmatic clinical trial of hearing screening in primary care clinics: cost-effectiveness of hearing screening. Cost Eff Resour Alloc..

[22] Deal J.A., Lin F.R. (2021). USPSTF Recommendations for Hearing Loss Screening in Asymptomatic Older Adults-A Case of Missing Evidence and Missing Treatment Options. JAMA Netw Open..

[23] Polspoel S., Moore D.R., Swanepoel D.W., Kramer S.E., Smits C. (2024). Global access to speech hearing tests. medRxiv..

